# Ethnoracial inequalities and child mortality in Brazil: a nationwide longitudinal study of 19 million newborn babies

**DOI:** 10.1016/S2214-109X(22)00333-3

**Published:** 2022-09-13

**Authors:** Poliana Rebouças, Emanuelle Goes, Julia Pescarini, Dandara Ramos, Maria Yury Ichihara, Samila Sena, Rafael Veiga, Laura C Rodrigues, Maurício L Barreto, Enny S Paixão

**Affiliations:** a Center for Data and Knowledge Integration for Health (CIDACS), Gonçalo Moniz Institute, Oswaldo Cruz Foundation, Salvador, Bahia, Brazil; b Instituto de Saude Coletiva, Federal University of Bahia, Salvador, Bahia, Brazil; c Faculty of Epidemiology and Population Health, London School of Hygiene & Tropical Medicine, London, UK; d Iyaleta Research Association-Research, Science and Humanities, Salvador, Bahia, Brazil

## Abstract

**Background:**

Racism is a social determinant of health inequities. In Brazil, racial injustices lead to poor outcomes in maternal and child health for Black and Indigenous populations, including greater risks of pregnancy-related complications; decreased access to antenatal, delivery, and postnatal care; and higher childhood mortality rates. In this study, we aimed to estimate inequalities in childhood mortality rates by maternal race and skin colour in a cohort of more than 19 million newborns in Brazil.

**Methods:**

We did a nationwide population-based, retrospective cohort study using linked data on all births and deaths in Brazil between Jan 1, 2012, and Dec 31, 2018. The data consisted of livebirths followed up to age 5 years, death, or Dec 31, 2018. Data for livebirths were extracted from the National Information System for livebirths, SINASC, and for deaths from the Mortality Information System, SIM. The final sample consisted of complete data for all cases regarding maternal race and skin colour, and no inconsistencies were present between date of birth and death after linkage. We fitted Cox proportional hazard regression models to calculate the crude and adjusted hazard ratios (HRs) and 95% CIs for the association between maternal race and skin colour and all-cause and cause-specific younger than age 5 mortality rates, by age subgroups. We calculated the trend of HRs (and 95% CI) by time of observation (calendar year) to indicate trends in inequalities.

**Findings:**

From the 20 526 714 livebirths registered in SINASC between Jan 1, 2012, and Dec 31, 2018, 238 436 were linked to death records identified from SIM. After linkage, 1 010 871 records were excluded due to missing data on maternal race or skin colour or inconsistent date of death. 19 515 843 livebirths were classified by mother's race, of which 224 213 died. Compared with children of White mothers, mortality risk for children younger than age 5 years was higher among children of Indigenous (HR 1·98 [95% CI 1·92–2·06]), Black (HR 1·39 [1·36–1·41]), and Brown or Mixed race (HR 1·19 [1·18–1·20]) mothers. The highest hazard ratios were observed during the post-neonatal period (Indigenous, HR 2·78 [95% CI 2·64–2·95], Black, HR 1·54 [1·48–1·59]), and Brown or Mixed race, HR 1·25 [1·23–1·27]) and between the ages of 1 year and 4 years (Indigenous, HR 3·82 [95% CI 3·52–4·15]), Black, HR 1·51 [1·42–1·60], and Brown or Mixed race, HR 1·30 [1·26–1·35]). Children of Indigenous (HR 16·39 [95% CI 12·88–20·85]), Black (HR 2·34 [1·78–3·06]), and Brown or Mixed race mothers (HR 2·05 [1·71–2·45]) had a higher risk of death from malnutrition than did children of White mothers. Similar patterns were observed for death from diarrhoea (Indigenous, HR 14·28 [95% CI 12·25–16·65]; Black, HR 1·72 [1·44–2·05]; and Brown or Mixed race mothers, HR 1·78 [1·61–1·98]) and influenza and pneumonia (Indigenous, HR 6·49 [95% CI 5·78–7·27]; Black, HR 1·78 [1·62–1·96]; and Brown or Mixed race mothers, HR 1·60 [1·51–1·69]).

**Interpretation:**

Substantial ethnoracial inequalities were observed in child mortality in Brazil, especially among the Indigenous and Black populations. These findings demonstrate the importance of regular racial inequality assessments and monitoring. We suggest implementing policies to promote ethnoracial equity to reduce the impact of racism on child health.

**Funding:**

MCTI/CNPq/MS/SCTIE/Decit/Bill & Melinda Gates Foundation's Grandes Desafios Brasil, Desenvolvimento Saudável para Todas as Crianças, and Wellcome Trust core support grant awarded to CIDACS-Center for Data and Knowledge Integration for Health.

## Introduction

Long-standing efforts to reduce child mortality intensified with the introduction of the Millennium and Sustainable Development Goals, but attention to ethnoracial inequalities has been limited.^1^, ^2^

The first steps in reducing ethnoracial inequalities are documenting evidence and recognising the root cause: systemic, structural, and institutionalised ethnoracism, which builds structures reflecting inequalities of power and values in a society. These structures include laws, policies, institutional practices, and ingrained beliefs that accept, promote, and maintain such inequalities. Furthermore, these inequalities establish unjust standards and solid foundations for their maintenance, including practices such as disenfranchisement, segregation, and mass incarceration of black people.^3^ Ethnoracism is a structural determinant of health,^4^ imposing barriers to accessing resources and opportunities in hiring, remuneration, adequate housing, and food security.^5^ Institutional racism imposes barriers to access and quality services, such as daycare centres, schools, and health care, including discriminatory practices within health services and other institutions.^6^ Effects of institutional racism influencing maternal and child health outcomes include the risks of pregnancy-related complications resulting in maternal or child health problems and deaths, barriers to access to antenatal, delivery, and postnatal care, and services which are unfavourable to Black, Brown or Mixed race, and Indigenous mothers and children in Brazil.^5^, ^7^, ^8^, ^9^, ^10^ Differences in the odds of child survival among different racial and ethnic groups were found when investigated in previous studies, with lower odds among Black, Brown or Mixed race, and Indigenous children.^11^, ^12^

**Research in context**

**Evidence before this study**

We searched PubMed to July 14, 2021 (without limiting by start date), using the search terms (Brazil) AND ((infant OR “under-five” OR child OR neonatal OR postneonatal) AND (mortality OR death)) AND (ethnicity OR race OR skin colour OR racial inequalit*), with a title and abstract restriction on the search terms, and no language restrictions. Our research only identified five articles on racial inequalities in infant mortality in Brazil, of which three were descriptive. The two papers that measured the association between race and skin colour and child mortality did not include the Indigenous population, and were from 2001 and 2008, used small samples from a restricted geographical area in Brazil, and did not investigate causes of death. There is a clear lack of literature on ethnoracial inequalities in childhood mortality in Brazil, despite the UN Sustainable Development Goal 17.18 recommendation of disaggregating indicators according to race and skin colour.

**Added value of this study**

Our analyses included more than 19 million livebirths nationwide between 2012 and 2018. The births were linked with all deaths of children younger than age 5 years in the same period. We found significant differences in the risk of death by race and skin colour among children younger than age 5 years and in all age groups (neonatal, post-neonatal, and children aged 1–4 years). The increase in mortality was highest in the post-neonatal period and at ages 1–4 years. When broken down by cause, racial inequalities were more marked on poverty-related causes such as malnutrition and diarrhoea. Our findings provide a better understanding of mortality inequalities by ethnicity in Brazil, identifying substantial disparities between groups by age and cause of death.

**Implications of all the evidence available**

Given that this comprehensive and current analysis of ethnoracial inequalities in child mortality identified marked differentials, we suggest that regular analyses of ethnic disparities in children younger than age 5 years should be done to monitor trends and direct health interventions. These analyses will not only allow international comparisons, but also enable an efficient assessment of the effect of existing policies.

In Brazil, extreme inequality is driven by class, gender, and race or ethnicity,^5^ and ethnoracial inequalities in child survival have been documented between 1950 and 2000.^13^, ^14^ No nationally representative study of ethnoracial inequalities in child survival has been identified after this period. This analysis aimed to examine childhood mortality rates according to maternal race or skin colour in a recent 6-year follow-up of a cohort of more than 19 million Brazilian children, in an effort to inform political decisions and public health interventions to reduce ethnoracial inequality in child health.

## Methods

### Study design and population

We did a nationwide retrospective cohort study, including all livebirths in Brazil, linked to mortality data from Jan 1, 2012, to Dec 31, 2018. The data consisted of livebirths followed up to age 5 years, death, or Dec 31, 2018, whichever came first. The Brazilian Ministry of Health made available data on livebirths (National Information System for live births, SINASC) and on deaths (Mortality Information System, SIM). SINASC contains all Declarations of Live Births, a legal document completed by the health professional who assists with delivery, and has a record of 98% of all livebirths in Brazil.^15^ The Declaration of Live Births includes maternal information (maternal age, schooling, marital status, and ethnicity); pregnancy information (antenatal appointments, length of gestation, or multiple fetuses); and information on the newborn such as birthweight and sex.^16^ SIM is a record of all death certificates with individual's name, date of birth, age, years of education, cause and date of death, mother's name and date of birth, and the municipality of residence. In 2011, SIM contained a record of 97% of all deaths in Brazil.^16^, ^17^, ^18^, ^19^ The final sample included all cases with complete information for maternal race and skin colour and no inconsistencies in dates of birth and death. Ethics approval was obtained from the Federal University of Bahia's Institute of Collective Health Ethics Committee (CAAE registration number, 180223194.00005.030).

### Procedures and outcomes

The accepted ethnicity terminology in Brazilian official records is race or skin colour. Maternal race or skin colour is the main exposure variable and these data were obtained from SINASC. Since 2011, a mother registering a child's birth has the option to classify herself as White, of Asian descent, Black, Brown or Mixed race, or Indigenous.^20^ This analysis used White as a reference category since the offspring off these women have had better overall health results.^21^, ^22^ Congenital anomalies were not excluded from the analysis.

The primary outcome of the study was child mortality, by age group and cause of death. Age groups were classified into neonatal (birth–27 days), post-neonatal (283–64 days), infant (birth–364 days), between the ages of 1 and 5, and younger than age 5 years (birth–5 years). Causes of death were classified according to the International Classification of Diseases 10th Revision, and the main poverty-related causes were selected (appendix p 1). We grouped deaths by accidental falls and drownings into a single group of causes (selected accidental causes), due to the small number of deaths and our understanding of their similar context.

We performed record linkage between SINASC and SIM in order to obtain our study population. The linkage process was based on a set of attributes: maternal name and age, date of birth, sex, and place of residence (municipality). We were not able to use the Live Birth Certificate number as a linkage attribute as it is only available for infant deaths and has a very low completion rate.^16^, ^21^ The linkage was performed by similarity matching, using the Center for Data and Knowledge Integration for Health-Record Linkage (CIDACS-RL) algorithm,^21^ an open-source linkage algorithm from CIDACS that generates a similarity score on the basis of several identifiers. The process was conducted in the CIDACS secure data centre environment, in a strict data protection set-up following ethical and legal standards.^22^ CIDACS-RL applies a combination of indexing and search algorithms. The indexing searches for the most similar records from the indexed SIM for each SINASC record and submits them to a paired comparison step. Candidate linking records are sorted according to their scores, and the highest-scoring comparison pair is retained. For this dataset, a sample of 2000 pairs, stratified into three linkage score categories (high score, more than 0·95; intermediate score, values between 0·90 and 0·95; and low score, less than 0·90) was manually reviewed to assess link quality. In this validation process, overall sensitivity and specificity was more than 90% in all years, with little variation (for example, 96·7% in 2001, 91·3% in 2008, and 94·0% in 2015 (appendix p 3).

### Statistical analysis

Descriptive statistics are presented for maternal sociodemographic data and newborn characteristics. Mortality rates by maternal race or skin colour were estimated (all causes and cause-specific, deaths per 100 000 person-years at risk), and crude hazard ratios (HRs) and 95% CIs were estimated using the Cox proportional hazard regression model. We calculated the trend of HRs (and 95% CIs) by time of observation (calendar year) to indicate a reduction or worsening of inequalities in mortality for those younger than age 5 years, by maternal race or skin colour. We present two analyses, a crude model and an adjusted model, using Cox regression for both. The adjusted models were estimated, controlling for the following variables: maternal education, region of residence at birth (proxy for poverty) and the child's year of birth (due to the impact of the Zika virus epidemic on birth rates). Nelson-Aalen estimates of cumulative hazard were calculated to compare mortality in the first 5 years of life by maternal race or skin colour. The Nelson-Aalen curve enables the comparison of populations through their cumulative risk curves.^23^ In our study, we preferred to use this curve instead of the Kaplan-Meier curve because of the relatively low number of events in our study population, and because it shows the risk of death for each ethnic group during follow-up time and not survival. We applied finite population correction to the crude and adjusted Cox models, according to age group. We graphically tested assumptions of proportionality of hazards. Missing values were excluded from adjusted analyses. The results of this study are reported in accordance with the REporting of studies Conducted using Observational Routinely-collected health Data guidelines (appendix p 5). Data analyses were done with Stata (version 15.0).

### Role of the funding source

The study's funders had no role in the study design, data collection, data analysis, data interpretation, writing of the report, or the decision to submit for publication.

## Results

**Figure 1:**
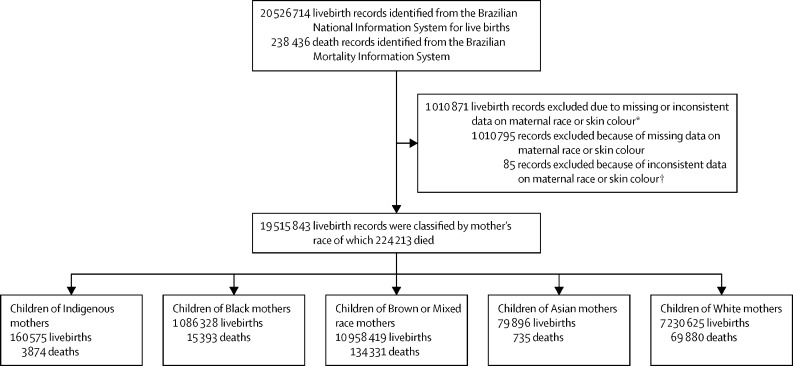
Study profile *The reasons for exclusion do not add up to 1 010 880 because a missing record of the mother's race or skin colour might have occured in several individuals, including the 85 excluded for inconsistent data. †Contradictory data, such as negative age of death after linkage.

During the study period from Jan 1, 2012, to Dec 31, 2018, 20 526 714 livebirths were registered and, of this total, 1 010 871 (4·9%) were excluded because of absence of information on maternal race or skin colour or inconsistent data (eg, date of death recorded as prior to birth; figure 1). 19 515 843 livebirths were classified by mother's race, of which 224 213 died. 10 958 419 (56·2%) of 19 515 843 livebirths in Brazil during the study period were to Brown or Mixed race mothers. The second largest proportion of births was to White mothers (7 230 625 [37·1%]), followed by Black mothers (1 086 328 [5·6%]), Indigenous mothers (160 575 [0·8%]), and Asian mothers (79 896 [0·4%]; table 1).

**Table 1:** Distribution of maternal and child characteristics according to maternal race or skin colour

	**Children of White mothers (n=7 230 625)**	**Children of Indigenous mothers (n=160 575)**	**Children of Black mothers (n=1 086 328)**	**Children of Brown or Mixed race mothers (10 958 419)**	**Children of Asian descent mothers (n=79 896)**	**Total (n=19 515 843)**
**Region**						
North	181 330 (2·5%)	86 167 (53·7%)	49 581 (4·6%)	1 805 340 (16·5%)	4761 (6·0%)	2 127 179 (10·9%)
Northeast	687 819 (9·5%)	25 031 (15·6%)	285 799 (26·3%)	4 320 602 (39·4%)	17 448 (21·8%)	5 336 699 (27·3%)
Southeast	3 714 727 (51·3%)	13 020 (8·1%)	574 245 (52·9%)	3 483 230 (31·8%)	42 158 (52·8%)	7 827 380 (40·1%)
South	2 201 290 (30·4%)	10 067 (6·3%)	115 132 (10·6%)	369 398 (3·4%)	5738 (7·2%)	2 701 625 (13·8%)
Midwest	444 147 (6·1%)	26 072 (16·2%)	61 520 (5·7%)	977 986 (8·9%)	9758 (12·2%)	1 519 483 (7·8%)
Missing	1312 (<0·1%)	218 (0·1%)	51 (<0·1%)	1863 (<0·1%)	33 (<0·1%)	3477 (<0·1%)
**Mother's years of education**						
3 years or less	104 785 (1·4%)	39 327 (24·5%)	50 960 (4·7%)	484 658 (4·4%)	1870 (2·3%)	681 600 (3·5%)
4–7 years	850 810 (11·8%)	49 564 (30·9%)	247 523 (22·8%)	2 499 434 (22·8%)	10 222 (12·8%)	3 657 553 (18·7%)
8 years or more	6 216 006 (86·0%)	63 867 (39·8%)	774 785 (71·3%)	7 789 753 (71·1%)	66 612 (83·4%)	14 911 023 (76·4%)
Missing	59 024 (0·8%)	7817 (4·9%)	13 060 (1·2%)	184 574 (1·7%)	1192 (1·5%)	265 667 (1·4%)
**Mother's age, years**						
Younger than 20	928 622 (12·8%)	46 688 (29·1%)	183 919 (16·9%)	2 309 744 (21·1%)	10 531 (13·2%)	3 479 504 (17·8%)
20–34	5 092 362 (70·4%)	96 659 (60·2%)	754 171 (69·4%)	7 438 819 (67·9%)	54 304 (68·0%)	13 436 315 (68·8%)
35 or older	1 189 207 (16·4%)	16 248 (10·1%)	145 630 (13·4%)	1 179 507 (10·8%)	14 850 (18·6%)	2 545 442 (13·0%)
Missing	20 434 (0·3%)	980 (0·6%)	2608 (0·2%)	30 349 (0·3%)	211 (0·3%)	54 582 (0·3%)
**Mother's marital status**						
Married or stable union	4 498 106 (62·2%)	87 376 (54·4%)	502 305 (46·2%)	5 836 181 (53·3%)	49 316 (61·7%)	10 973 284 (56·2%)
Single	2 573 758 (35·6%)	68 960 (42·9%)	563 342 (51·9%)	4 929 546 (45·0%)	28 409 (35·6%)	8 164 015 (41·8%)
Separated or divorced or widowed	126 788 (1·8%)	873 (0·5%)	12 909 (1·2%)	110 118 (1·0%)	1654 (2·1%)	252 342 (1·3%)
Missing	31 973 (0·4%)	3366 (2·1%)	7772 (0·7%)	82 574 (0·8%)	517 (0·6%)	126 202 (0·6%)
**Number of living children**						
None	3 408 683 (47·1%)	40 645 (25·3%)	408 779 (37·6%)	4 057 494 (37·0%)	36 289 (45·4%)	7 951 890 (40·7%)
1	2 351 254 (32·5%)	34 713 (21·6%)	316 922 (29·2%)	3 312 300 (30·2%)	25 895 (32·4%)	6 041 084 (31·0%)
2	838 548 (11·6%)	24 922 (15·5%)	164 715 (15·2%)	1 625 165 (14·8%)	9398 (11·8%)	2 662 748 (13·6%)
3 or more	426 018 (5·9%)	54 500 (33·9%)	149 543 (13·8%)	1 299 163 (11·9%)	5073 (6·3%)	1 934 297 (9·9%)
Missing	206 122 (2·9%)	5795 (3·6%)	46 369 (4·3%)	664 297 (6·1%)	3241 (4·1%)	925 824 (4·7%)
**Prenatal consultations**						
3 or less	343 149 (4·7%)	45 848 (28·6%)	117 498 (10·8%)	1 165 540 (10·6%)	5602 (7·0%)	1 677 637 (8·6%)
4–6	1 237 214 (17·1%)	61 687 (38·4%)	289 707 (26·7%)	3 146 867 (28·7%)	17 067 (21·4%)	4 752 542 (24·4%)
7 or more	5 610 661 (77·6%)	51 324 (31·9%)	669 639 (61·6%)	6 568 118 (59·9%)	56 800 (71·1%)	12 956 542 (66·4%)
Missing	39 601 (0·5%)	1716 (1·1%)	9484 (0·9%)	77 894 (0·7%)	427 (0·5%)	129 122 (0·7%)
**Mode of delivery**						
Vaginal birth	2 380 803 (32·9%)	128 149 (79·8%)	551 200 (50·7%)	5 480 527 (50·0%)	33 723 (42·2%)	8 574 402 (43·9%)
Caesarean section	4 844 237 (67·0%)	31 911 (19·9%)	533 783 (49·1%)	5 463 058 (49·9%)	46 070 (57·7%)	10 919 059 (55·9%)
Missing	5585 (<0·1%)	515 (0·3%)	1345 (0·1%)	14 834 (0·1%)	103 (0·1%)	22 382 (0·1%)
**Gestational age, weeks**						
<37	809 122 (11·2%)	23 509 (14·6%)	129 602 (11·9%)	1 222 099 (11·2%)	9040 (11·3%)	2 193 372 (11·2%)
≥37	6 327 599 (87·5%)	127 198 (79·2%)	931 893 (85·8%)	9 379 057 (85·6%)	69 404 (86·9%)	16 835 151 (86·3%)
Missing	93 904 (1·3%)	9868 (6·1%)	24 833 (2·3%)	357 263 (3·3%)	1452 (1·8%)	487 320 (2·5%)
**Birthweight, kg**						
<2·5	621 537 (8·6%)	12 618 (7·9%)	107 440 (9·9%)	903 501 (8·2%)	7186 (9·0%)	1 652 282 (8·5%)
≥2·5	6 609 088 (91·4%)	147 957 (92·1%)	978 888 (90·1%)	10 054 918 (91·8%)	72 710 (91·0%)	17 863 561 (91·5%)
Missing	0	0	0	0	0	0
**Child's gender**						
Female	3 536 613 (48·9%)	78 203 (48·7%)	526 773 (48·5%)	5 338 555 (48·7%)	39 005 (48·8%)	9 519 149 (48·8%)
Male	3 693 022 (51·1%)	82 341 (51·3%)	559 339 (51·5%)	5 617 792 (51·3%)	40 884 (51·2%)	9 993 378 (51·2%)
Missing	990 (<0·1%)	31 (<0·1%)	216 (<0·1%)	2072 (<0·1%)	7 (<0·1%)	3316 (<0·1%)

Data are n (%).

Most births occurred in the southeast region of the country (7 827 405 [40·1%]), followed by the northeast region (5 336 730 [27·3%]). However, most Indigenous mothers gave birth in the north of the country (86 168 [53·7%] of 160 575). Indigenous, Black, and Brown or Mixed race mothers had fewer years of schooling and were younger than White mothers (table 1). Many Black women were single mothers (563 342 [51·9%] of 1 086 328), but there was also a higher proportion of single mothers among Indigenous (68 960 [42·9%] of 160 575) and Brown or Mixed race women (4 929 546 [45·0%] of 10 958 419) than White women (2 573 758 [35·6%] of 7 230 652). The proportion of mothers with three or more children was higher among Indigenous (33·9%), Black (13·7%), and Brown or Mixed race women (11·9%) than among White women (5·9%; table 1).

Regarding health care, a lower proportion of White mothers (4·7%) had fewer than three prenatal consultations than did Indigenous (28·6%), Black (10·8%), and Brown or Mixed race mothers (10·6%). The proportion of caesarean sections was 67·0% among livebirths to White mothers, while among Indigenous mothers, most deliveries were vaginal (79·8%; table 1). Regarding pregnancy outcomes, almost 15% of all Indigenous livebirths were premature (ie, born at <37 weeks’ gestation), which was higher than the proportion among White mothers (11·2%). In all ethnoracial groups, at least 90% of newborn babies had a birthweight of 2·5 kg or more (table 1).

Mortality per 100 000 person-years at risk along with HRs and 95% CIs for the comparison of mortality between ethnic groups are shown in table 2. A higher mortality per 100 000 person-years at risk was observed in all age groups among children of Indigenous (neonatal, 14 051·00 [95% CI 13 399·00–14 734·00]; post-neonatal, 1091 [1037·00–1149·00]; and age 1–4, 207·77 [193·08–223·57]), followed by Black (neonatal, 11 661·00 [95% CI 11 430·00–11 897·00]; post-neonatal: 469·69 [455·82–483·97]; and age 1–4: 63·28 [60·19–66·54]), and Brown or Mixed race mothers (neonatal, 10 305·00 [95% CI 10 237·00–10 375·00]; post-neonatal, 380·85 [376·89–384·85); and age 1–4: 55·19 [54·27–56·13]), than was observed in White mothers (neonatal, 8451·00 [95% CI 8374·00–8528·00]; post-neonatal, 279·00 [274·88–283·23]; and age 1–4: 38·24 [37·31–39·19]; table 2).

**Table 2:** Crude and adjusted HRs for the association between maternal race and skin colour and mortality younger than age 5 years, by age group

	**Children of White mothers**	**Children of Indigenous mothers**	**Children of Black mothers**	**Children of Brown or Mixed race mothers**	**Children of Asian descent mothers**
**Younger than age 5 years**					
Participants	69 880/224 213 (31·2%)	3874/224 213 (1·7%)	15 393/224 213 (6·9%)	134 331/224 213 (59·9%)	735/224 213 (0·3%)
Deaths per 100 000 person-years at risk (95% CI)	300·40 (298·18–302·64)	792·51 (767·94–817·86)	452·32 (445·23–459·52)	388·63 (386·56–390·72)	293·39 (272·92–315·38)
HR (95% CI)	1 (ref)	2·53 (2·45–2·61)	1·48 (1·45–1·50)	1·27 (1·26–1·28)	0·96 (0·89–1·03)
Adjusted HR (95% CI)	1 (ref)	1·98 (1·92–2·06)	1·39 (1·36–1·41)	1·19 (1·18–1·20)	0·86 (0·80–0·93)
**Age 1–4 years**					
Participants	6345/22 198 (28·9%)	715/22 198 (3·2%)	1528/22 198 (6·9%)	13 539/22 198 (61·0%)	71/22 198 (0·3%)
Deaths per 100 000 person-years at risk (95% CI)	38·24 (37·31–39·19)	207·77 (193·08–223·57)	63·28 (60·19–66·54)	55·19 (54·27–56·13)	39·96 (31·67–50·43)
HR (95% CI)	1 (ref)	5·40 (5·00–5·83)	1·65 (1·56–1·74)	1·43 (1·39–1·48)	1·04 (0·82–1·32)
Adjusted HR (95% CI)	1 (ref)	3·82 (3·52–4·15)	1·51 (1·42–1·60)	1·30 (1·26–1·35)	1·00 (0·79–1·27)
**Younger than age 1 year**					
Participants	63 535/202 012 (31·5%)	3159/202 012 (1·6%)	13 864/202 012 (6·9%)	120 790/202 012 (59·8%)	664/202 012 (0·3%)
Deaths per 100 000 person-years at risk (95% CI)	950·98 (943·61–958·40)	2179·00 (2104·00–2256·00)	1399·00 (1376·00–1423·00)	1201·00 (1194·00–1208·00)	909·69 (843·07–981·58)
HR (95% CI)	1 (ref)	2·25 (2·17–2·33)	1·45 (1·43–1·48)	1·25 (1·24–1·26)	0·94 (0·87–1·02)
Adjusted HR (95% CI)	1 (ref)	1·79 (1·73–1·87)	1·37 (1·35–1·40)	1·18 (1·17–1·19)	0·93 (0·86–1·00)
**Post-neonatal (28–364 days)**					
Participants	17 165/58 344 (29·4%)	1455/58 344 (2·5%)	4280/58 344 (7·3%)	35 247/58 344 (60·4%)	197/58 344 (0·3%)
Deaths per 100 000 person-years at risk (95% CI)	279·02 (274·88–283·23)	1091·00 (1037·00–1149·00)	469·69 (455·82–483·97)	380·85 (376·89–384·85)	293·38 (255·15–337·35)
HR (95% CI)	1 (ref)	3·88 (3·68–4·10)	1·68 (1·62–1·73)	1·36 (1·34–1·39)	1·05 (0·91–1·20)
Adjusted HR (95% CI)	1 (ref)	2·78 (2·64–2·95)	1·54 (1·48–1·59)	1·25 (1·23–1·27)	1·03 (0·90–1·19)
**Neonatal (from birth to 27 days)**					
Participants	46 370/143 668 (32·3%)	1704/143 668 (1·2%)	9584/143 668 (6·7%)	85 543/143 668 (59·5%)	467/143 668 (0·3%)
Deaths per 100 000 person-years at risk (95% CI)	8451·00 (8374·00–8528·00)	14 051·00 (13 399·00–14 734·00)	11 661·00 (11 430·00–11 897·00)	10 305·00 (10 237·00–10 375·00)	7707·00 (7039·00–8439·00)
HR (95% CI)	1 (ref)	1·65 (1·58–1·74)	1·37 (1·34–1·40)	1·21 (1·20–1·23)	0·91 (0·83–0·99)
Adjusted HR (95% CI)	1 (ref)	1·38 (1·31–1·45)	1·31 (1·28–1·34)	1·16 (1·14–1·17)	0·89 (0·82–0·98)

Data are n/N (%) or HR (95% CI), unless otherwise specified. Model adjusted for the variables: region, mother's education, and year of birth. HR=hazard ratio.

In crude analyses, children of Indigenous mothers were twice as likely to die before age 5 years as were White children (HR 2·53 [95% CI 2·45–2·61]). Among children born to Black and Brown/Mixed race mothers, the probability of death was 48% and 27% higher than among children of White mothers (HR 1·48 [95% CI 1·45–1·50 and HR 1·27 [1·26–1·28]) when compared with children of White mothers. In the group of children aged 1–4 years, mortality was 5 times higher among children of Indigenous mothers than among those of White mothers (HR 5·40 [95% CI 5·00–5·83]). Furthermore, mortality in children aged 1–4 years was 65% higher among children of Black mothers (HR 1·65 [95% CI 1·56–1·74]) and 43% higher among children of Brown or Mixed race mothers (HR 1·43 [1·39–1·48]). Among the post-neonates, the probability of death remained higher among children of Indigenous, Black, and Brown or Mixed race mothers than for children of White mothers (Indigenous, HR 3·88 [95% CI 3·68–4·10]; Black, HR 1·68 [1·62–1·73]; and Brown or Mixed race, HR 1·36 [1·34–1·39]), and the same was observed among newborn children (children of Indigenous mothers, HR 1·65 [95% CI 1·58–1·74]; Black mothers, HR 1·37 [1·34–1·40]; and Brown or Mixed race mothers, HR 1·21 [1·20–1·23]; table 2).

The association between maternal race and skin colour and mortality younger than age 5 years was lower after adjustment than in the crude model but remained significant. The children of Indigenous mothers were 98% (HR 1·98 [95% CI 1·92–2·06]) more likely to die in the first 5 years of life than those of White mothers. The mortality rate was 39% and 19% higher among children with Black and Brown/Mixed race mothers (HR 1·39 [95% CI 1·36–1·41] and HR 1·19 [1·18–1·20) than for White mothers (table 2). In the age group of 1–4 years, following adjustment, mortality was 3·8 times higher (HR 3·82 [95% CI 3·52–4.15]) among children of Indigenous mothers than of White mothers, and 51% higher for children of Black mothers (HR 1·51 [1·42–1·60]) and 30% higher for children of Brown or Mixed race mothers (HR 1·30 [1·26–1·35]; table 2).

The strongest estimates of association between maternal race or skin colour and child mortality were found for the HRs of influenza and pneumonia (table 3). Crude analyses show that among children of Indigenous mothers, the probability of death from diarrhoea, malnutrition, and influenza and pneumonia was 31, 11, and 52 times higher than among children of White mothers (diarrhoea, HR 31·62 [95% CI 27·66–36·14]; influenza and pneumonia, HR 11·69 [10·55–12·97]; malnutrition, HR 52·82 [42·83–65·14]). Among children of Black mothers, mortality in children younger than 5 years from these causes was about two times higher for diarrhoea (HR 2·19 [95% CI 1·84–2·60]) and influenza and pneumonia (HR 2·11 [1·92–2·32]) and about three times higher for malnutrition (HR 3·46 [2·66–4·50]) compared with children of White mothers. For children of Brown or Mixed race mothers, mortality was about two times higher for diarrhoea (HR 2·40 [95% CI 2·88–2·65]) and influenza and pneumonia (HR 1·98 [1·87–2·08]) and about three times higher for malnutrition (HR 3·30 [2·79–3·90]) than observed for children of White mothers. There was also a higher risk of death from selected accidental causes among children of Indigenous, Black, and Brown or Mixed race mothers (Indigenous, HR 4·58 [95% CI 3·44–6·11]; Black, HR 1·59 [1·31–1·94]; Brown or Mixed race, HR 1·61 [1·46–1·78) than among children of White mothers. Risk of death from ill-defined causes was eight times higher among children of Indigenous women (HR 8·08 [95% CI 7·05–9·26) and about two and three times higher among children of Brown or Mixed race women (HR 1·93 [1·81–2·05]) and Black women (HR 2·73 [2·48–3·01]) than among children of White women (table 3).

**Table 3:** Crude and adjusted HRs for the association between maternal race and skin colour and mortality younger than age 5 years, according to the main causes of death

	**Children of White mothers**	**Children of Indigenous mothers**	**Children of Black mothers**	**Children of Brown or Mixed race mothers**	**Children of Asian descent mothers**
**Diarrhoea**					
Participants	528/2971 (17·8%)	361/2971 (12·2%)	171/2971 (5·8%)	1906/2971 (64·2%)	5/2971 (0·2%)
Deaths per 100 000 person-years at risk (95% CI)	2·27 (2·08–2·47)	73·85 (66·61–81·88)	5·02 (4·33–5·84)	5·51 (5·27–5·77)	1·99 (0·83–4·79)
HR (95% CI)	1 (ref)	31·62 (27·66–36·14)	2·19 (1·84–2·60)	2·40 (2·18–2·65)	0·87 (0·36–2·09)
Adjusted HR (95% CI)	1 (ref)	14·28 (12·25–16·65)	1·72 (1·44–2·05)	1·78 (1·61–1·98)	0·81 (0·33–1·94)
**Influenza and pneumonia**					
Participants	1787/8118 (22·0%)	450/8118 (5·5%)	557/8118 (6·9%)	5297/8118 (65·3%)	27/8118 (0·3%)
Deaths per 100 000 person-years at risk (95% CI)	7·68 (7·33–8·04)	92·06 (83·93–100·97)	16·37 (15·06–17·78)	15·32 (14·91–15·74)	10·77 (7·39–15·71)
HR (95% CI)	1 (ref)	11·69 (10·55–12·97)	2·11 (1·92–2·32)	1·98 (1·87–2·08)	1·39 (0·95–2·03)
Adjusted HR (95% CI)	1 (ref)	6·49 (5·78–7·27)	1·78 (1·62–1·96)	1·60 (1·51–1·69)	1·32 (0·91–1·94)
**Malnutrition**					
Participants	164/1250 (13·1%)	187/1250 (15·0%)	84/1250 (6·7%)	813/1250 (65·0%)	2/1250 (0·2%)
Deaths per 100 000 person-years at risk (95% CI)	0·71 (0·60–0·82)	38·25 (33·14–44·15)	2·47 (1·99–3·05)	2·35 (2·19–2·51)	0·80 (0·20–3·05)
HR (95% CI)	1 (ref)	52·82 (42·83–65·14)	3·46 (2·66–4·50)	3·30 (2·79–3·90)	1·12 (0·28–4·51)
Adjusted HR (95% CI)	1 (ref)	16·39 (12·88–20·85)	2·34 (1·78–3·06)	2·05 (1·71–2·45)	0·98 (0·24–3·94)
Selected accidental causes*					
Participants	531/1985 (26·8%)	51/1985 (2·6%)	124/1985 (6·2%)	1274/1985 (64·2%)	5/1985 (0·3%)
Deaths per 100 000 person-years at risk (95% CI)	2·28 (2·09–2·48)	10·43 (7·92–13·72)	3·64 (3·05–4·34)	3·68 (3·48–3·89)	1·99 (2·09–2·48)
HR (95% CI)	1 (ref)	4·58 (3·44–6·11)	1·59 (1·31–1·94)	1·61 (1·46–1·78)	0·87 (0·36–2·11)
Adjusted HR (95% CI)	1 (ref)	1·74 (1·27–2·37)	1·37 (1·12–1·67)	1·17 (1·04–1·32)	0·82 (0·34–1·99)
**Ill-defined causes**					
Participants	1382/6211 (22·3%)	242/6211 (3·9%)	560/6211 (9·0%)	4007/6211 (64·5%)	20/6211 (0·3%)
Deaths per 100 000 person-years at risk (95% CI)	5·94 (5·63–6·26)	49·51 (43·64–56·15)	16·45 (15·14–17·88)	11·59 (11·24–11·96)	7·98 (5·15–12·37)
HR (95% CI)	1 (ref)	8·08 (7·05–9·26)	2·73 (2·48–3·01)	1·93 (1·81–2·05)	1·32 (0·85–2·06)
Adjusted HR (95% CI)	1 (ref)	4·26 (3·67–4·94)	2·29 (2·07–2·53)	1·58 (1·48–1·68)	1·29 (0·83–2·00)

Data are n/N (%) or HR (95% CI), unless otherwise specified. Model adjusted for the variables: region, mother's education, and year of birth. HR=hazard ratio.

* The selected accidental causes refer to deaths from drowning and falls.

Adjusted younger than age 5 mortality rates for diarrhoea, malnutrition, and pneumonia, were 14·28, 16·39, and 6·49 times higher among children of Indigenous mothers than those of White mothers (diarrhoea, HR 14·28 [95% CI 12·25–16·65]; malnutrition, HR 16·39 [12·88–20·85]; influenza and pneumonia, HR 6·49 [5·78–7·27]). For children of Black mothers, the younger than age 5 mortality rate for these causes was 72%, 2·34 times, and 78% higher than those for White mothers (HR 1·72 [95% CI 1·44–2·05]; HR 2·34 [1·78–3·06]; and HR 1·78 [1·62–1·96]) and for Brown or Mixed race mothers was 78%, 2·05 times, and 60% (diarrhoea, HR 1·78 [95% CI 1·61–1·98]; malnutrition, HR 2·05; [1·71–2·45]; influenza and pneumonia, HR 1·60 [1·51–1·69]) higher than among children of White mothers (table 3). Children of Indigenous and Black mothers had higher risk of death from selected accidental causes (HR 1·74 [95% CI 1·27–2·37] and HR 1·37 [1·12–1·67]) than did children of White mothers. Increased mortality risk for ill-defined causes was also observed among Indigenous (HR 4·26 [95% CI 3·67–4·94), Black (HR 2·29 [2·07–2·53]), and Brown or Mixed race children (HR 1·58 [1·48–1·68]) compared with those of White mothers**.**

The association between maternal race and skin colour and cause-specific child mortality by age group (neonatal mortality and deaths from age 1 to 4 years) is described in the appendix (pp 10–11). The tables included in the appendix show the same pattern of racial inequality in mortality by cause in each age group, with greater magnitude among children aged 1–4 years.

The cumulative risk of death before the age of 5 years was higher among children of Indigenous mothers throughout the follow-up period than in other groups, especially when compared with the group of children of White mothers or mothers of Asian descent, the ones with the lowest younger than age 5 cumulative mortality risk (appendix p 12). After Indigenous children, children of Black mothers had the greatest risk of mortality before age 5 years throughout the study period.

**Figure 2:**
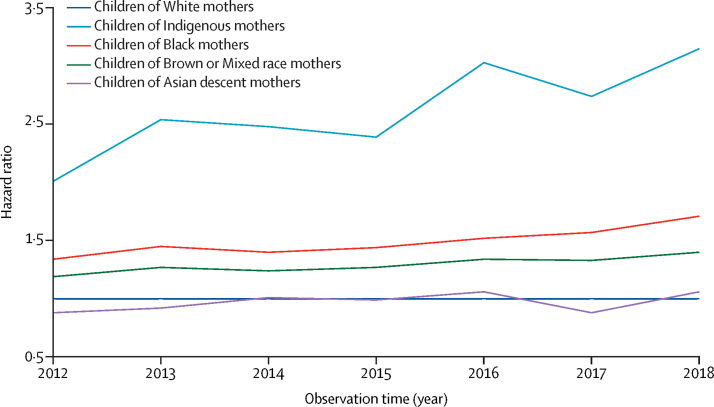
Trends in hazard ratio for mortality in children younger than 5 years, by calendar year

The graph by calendar year shows the trend of HRs over the observed time period (figure 2). The graph shows a trend towards increasing inequality for mortality in those aged younger than 5 years, especially among children of Indigenous and Black mothers compared with White mothers.

We applied finite population correction to the crude and adjusted Cox models, according to age group, and found that the confidence interval values did not change significantly. Therefore, we decided to keep the original results and included the table by age group with the finite population correction in the appendix (p 13). We graphically tested assumptions of proportionality of hazards and the curves indicated that the proportionality of hazards assumption was satisfied (appendix p 4).

## Discussion

Our results showed significant disparities in mortality rates among children from mothers of different ethnoracial groups in Brazil. Even after adjustment, we found that children of Indigenous mothers were 98% more likely to die than those of White mothers in the first 5 years of life. Mortality younger than age 5 years was 39% and 19% higher among children of Black and Brown or Mixed race mothers than children of White mothers. The largest ethnoracial disparities were observed in children aged 1–4 years for diarrhoea, malnutrition, and influenza and pneumonia.

Previous studies have also shown persistent ethnoracial disparities in child health outcomes in Brazil.^10^, ^24^, ^25^, ^26^, ^27^, ^28^, ^29^ However, very few specific studies on mortality and ethnoracial inequalities are available, and none have studied specific causes of death nor the 1–5 years age group, and most do not include the Indigenous population.^24^, ^25^

Similar to other Latin American countries, the Brazilian population originated from three main ancestral roots: African, European, and Native American, with Native American the original Indigenous population. Colonisation was predominantly by White Europeans (Portuguese in Brazil, and Spanish in other Latin American countries). The slave trade of Africans to Brazil was the highest in American and Caribbean countries. An estimated 3·6 million African slaves, 7 times more than their counterparts in the USA, were brought to Brazil.^30^ Over the centuries, structural racism has persisted and is expressed in economic injustice and social deprivation among the Black and Indigenous population, resulting in worse education, employment, and housing; lower wages; inadequate health care; psychosocial trauma; political exclusion; environmental inequalities; and other types of related violence.^6^ Childhood survival is directly linked to living conditions of parents or caregivers.^31^ Thus, the parents’ educational qualifications and occupational status, family structure, housing, income, and the socioeconomic characteristics of the neighbourhood in which they live will mediate social-related risks and exposures, contributing to health outcomes before and after birth. In this context, the burden of structural racism experienced by caregivers will be transmitted to the child, producing ethnoracial inequalities in health.^7^

Current studies show numerous ways that racism affects health and involves unfavourable physical, social, and economic exposures co-occurring, accumulated in the course of life, and transmitted between generations.^32^, ^33^ With economic injustice and social deprivation, Black, Brown or Mixed race, and Indigenous people will be subject to less access to and inadequate health care; psychosocial trauma; political exclusion; environmental inequities; worse education, employment, and housing; lower wages; and violence.^32^, ^33^

In Brazil, Black, Brown or Mixed race, and Indigenous mothers live in unfavourable circumstances with less education, decreased frequency or late start of prenatal care, and reside further away from health-care facilities during childbirth.^10^, ^24^, ^26^, ^27^ These living circumstances result in a higher risk of negative outcomes, such as low birthweight, being born small for gestational age, prematurity, and an increased incidence of preventable diseases, all of which increase the risk of child mortality.^24^, ^25^

The survival of post-neonates and children aged 1–4 years is most adversely affected by the family's living conditions, income, education, basic sanitation, access to clean water, and access to health services. By contrast, newborn survival is associated with genetic disorders, gestational conditions, and perinatal characteristics.^34^, ^35^ This is consistent with the high degree of ethnoracial inequities in the mortality of post-neonatal children and those aged 1–4 years in our study. They are the age groups most strongly affected by the disadvantaged social circumstances of their families, defined by structural racism.

In recent years, inequalities in mortality younger than age 5 years by race or maternal skin colour has increased as shown by the results of this study. These inequalities require urgent attention, especially in the context of the global health crisis of the COVID-19 pandemic, which directly exacerbated pre-existing inequalities in Brazil.^36^

High disparities were observed in deaths related to malnutrition, diarrhoea, and influenza and pneumonia, which can be partially explained by more impoverished living conditions^14^ and a worse nutritional status for Black, Brown or Mixed race, and Indigenous children.^24^, ^32^, ^37^ Children younger than age 1 year living in the country's poorest regions suffer more deaths due to diarrhoea, as these regions have the worst social (ie, average per capita income, gross domestic product, and human development index) and sanitation conditions.^37^ Diarrhoea and respiratory infections are the leading causes of hospital admission among Indigenous children.^28^ The increased risk of death from falls and drowning among children of Indigenous, Black, and Brown or Mixed race mothers should be part of the discussion of family living conditions,^38^ given the strong association between social class and poverty and childhood falls and drowning. Social class and poverty are structural determinants of health conditions, and they are determined to varying degrees by structural racism.^3^ All these causes of death are potentially preventable if attention and care by health services is received in a timely manner, which makes it essential to recognise racism as a determinant of the lack of access to health and care opportunities for Indigenous and Black populations.^39^

To our knowledge, this is the most extensive study of ethnoracial disparities in child mortality in Brazil. It uses a cohort of nationwide livebirths of sufficient completeness and coverage.^15^ Measurement of racism through the race and skin colour variable is complex, and can vary according to whether individuals can self-classify or be classified.^40^ However, the strong evidence of the effects of ethnoracial disparities on health is indisputable, even in studies that use race and skin colour self-classification variables as a proxy for racism.^40^

Our study has some limitations. Firstly, the linkage between SINASC and SIM did not identify 100% of the deaths for children younger than age 5 years. As a result, our cohort identified 81·15% of deaths among children younger than age 5 registered in Brazil in the same period. Although the quality of the data has been improving over the years, some regional disparities remain.^41^, ^42^ Poor data quality can limit linkage quality; under-reporting of deaths is still more prevalent in the most impoverished areas of the country,^43^ which are the areas with Indigenous communities. These issues with data quality can lead to underestimating mortality in Indigenous groups compared with other ethnoracial groups. We did not analyse whether the 18·85% of unlinked deaths were biased, and this is another limitation of our study. Finally, some estimates related to the cause of death from malnutrition are unreliable due to the very small size of the sample involved, especially among newborn babies, as was also the case with deaths from selected accidental causes.

Our objective was to present an overall picture of ethnoracial inequalities in child survival in Brazil. Our results demonstrate the importance of monitoring ethnoracial disparities in other maternal and child health outcomes and their determinants not only in Brazil but also in other countries. Additional research could investigate the drivers of inequality in different geographical regions to investigate similar or different patterns of ethnoracial inequalities.

Although the demands of social movements in Brazil have been long standing, pressure to include ethnoracial issues on the public health policy agenda is continuous. Efforts have focused on implementing the National Comprehensive Health Policy for Indigenous People since 2002,^44^ and the National Comprehensive Health Policy for the Black Population since 2006.^45^ However, limited resources and the recent reduction in political status of Indigenous, Black, and Brown and Mixed race people has resulted in an unfavourable reality towards decreasing ethnoracial health inequalities. The findings of this study can be added to other efforts to reinforce the need for equitable public health policies, which include an agenda prioritising Black, Brown or Mixed race, and Indigenous children's health.

## Data sharing

All data supporting this study were obtained from the Center for Data and Knowledge Integration for Health (CIDACS). Restrictions apply to access these data, which contain sensitive information, were licensed for exclusive use in the current study and, due to privacy regulations from the Brazilian Ethics Committee, are not openly available. Upon reasonable request and with express permission from CIDACS (mail to cidacs.curadoria@fiocruz.br) and approval from an ethics committee, controlled access to the data is possible.

## Declaration of interests

We declare no competing interests.
