# Supplementary material for: Ethnoracial inequalities and child mortality in Brazil: a nationwide longitudinal study of 19 million newborn babies
Source: Lancet Glob Health. 2022 Sep 13;10(10):e1453–62. doi: 10.1016/S2214-109X(22)00333-3 (PMC9638038; doi:10.1016/S2214-109X(22)00333-3)
Supplement: Supplementary appendix [file mmc1.pdf]

THE LANCET
Global Health

Supplementary appendix

This appendix formed part of the original submission and has been peer reviewed. We post it as supplied by the authors.

Supplement to: Rebouças P, Goes E, Pescarini J, et al. Ethnoracial inequalities and child mortality in Brazil: a nationwide longitudinal study of 19 million newborn babies. *Lancet Glob Health* 2022; **10:** e1453–62.

Supplementary material

Table of contents

Table S1. Classification of causes of death according to ICD 10 chapters. 1

Figure S1 - Linkage flowchart 2

Table S2. Exact values for sensitivity and specificity about linkage SIM X SINASC 3

Figure S2 - Test of assumption of proportionality of hazards 4

Table S3. Observational Routinely-Collected health Data (RECORD) guideline 5

Table S4. Crude and adjusted Hazard Ratio for the association between maternal race/skin colour and neonatal mortality, according to the main causes of death. 10

Table S5. Crude and adjusted Hazard Ratio for the association between maternal race/skin colour and 1 to 4 years old mortality, according to the main causes of death. 11

Figure S3 - Cumulative under-five mortality per 100 000 person-years at risk by maternal race/skin colour obtained by the Nelson-Aalen method 12

Table S6. Crude and adjusted Hazard Ratio for the association between maternal race/skin colour and mortality by age group, with a finite population correction. 13

Table S1. Classification of causes of death according to ICD 10 chapters.

Classification of causes of death	ICD10
**Diarrhoea**	A00-A09
**Flu and pneumonia**	J09-J18
	J20-J22
**Malnutrition**	E40-E46
	E50-E56
	E58-E54
**Selected accidental causes***	W56-W70
	W73, W74
	W00-W46
	W49
**Ill-defined causes**	R00-R99

*The selected accidental causes refer to deaths from drowning (W56-W70; W73, W74) and falls (W00-W46; W49).

Figure S1 - Linkage flowchart

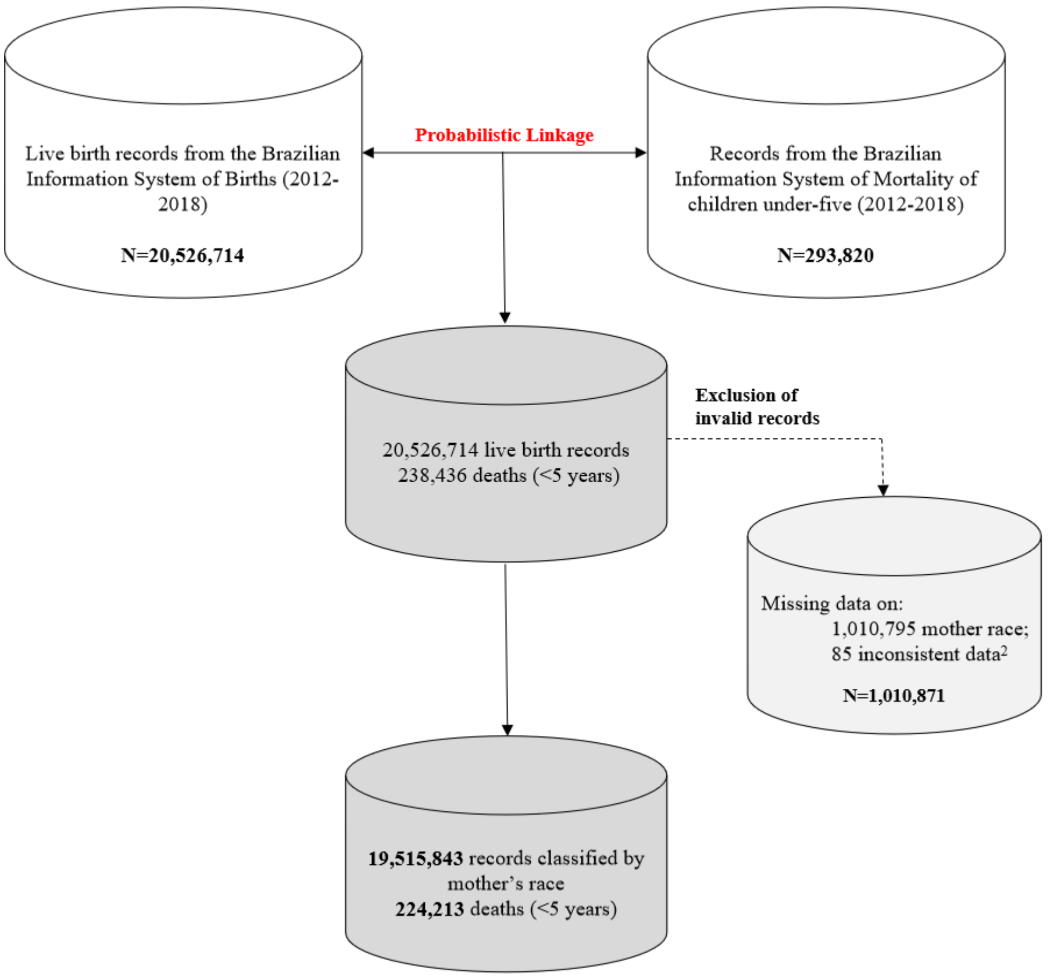

Table S2. Exact values for sensitivity and specificity about linkage SIM X SINASC

Year	Cutoff	Sensitivity (%)	Specificity (%)
**2012**	0.974	92.1	91.6
**2013**	0.974	93.3	94.3
**2014**	0.974	92.9	95.5
**2015**	0.973	94.0	94.0
**2016**	0.951	94.7	96.5
**2017**	0.950	95.0	98.1
**2018**	0.939	92.8	93.3

	Manual Review	
	**True**	**False**
**≥ Cutoff**	a	b
**> Cutoff**	c	d
	a+c	b+d

Sensitivity = a / (a+c)

Specificity = d / (b+d)

Figure S2 - Test of assumption of proportionality of hazards, crude (a) and adjusted (b) models

S2 a - Crude model

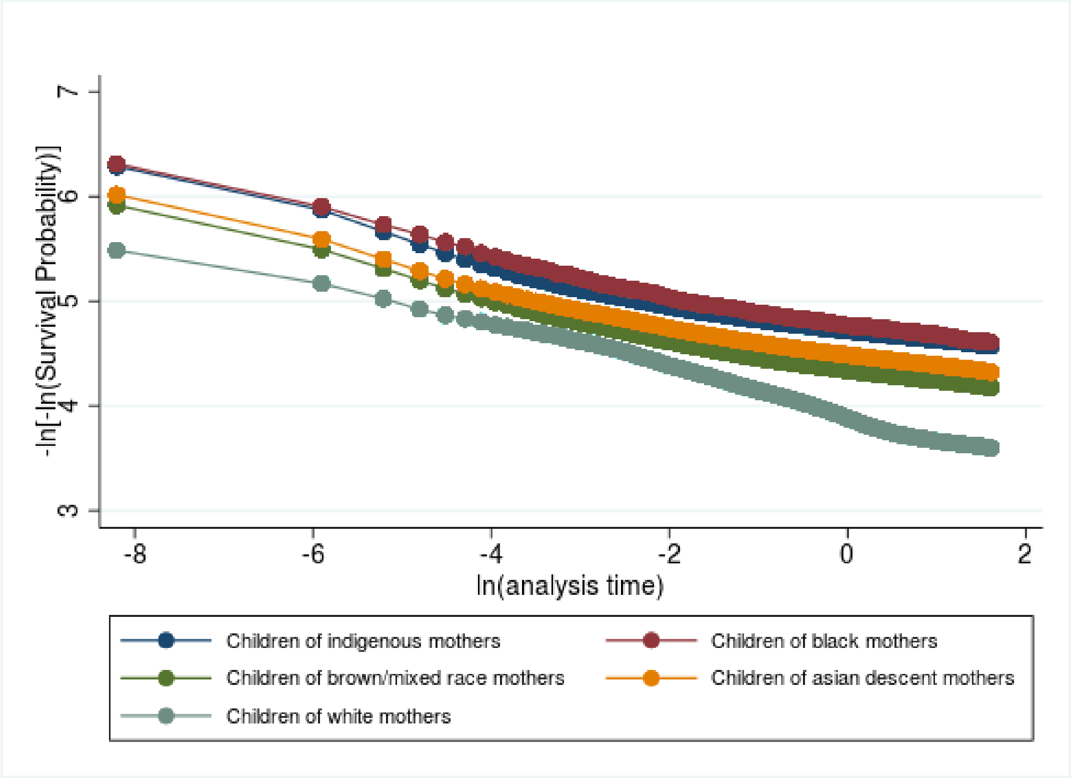

S2 b - Adjusted model

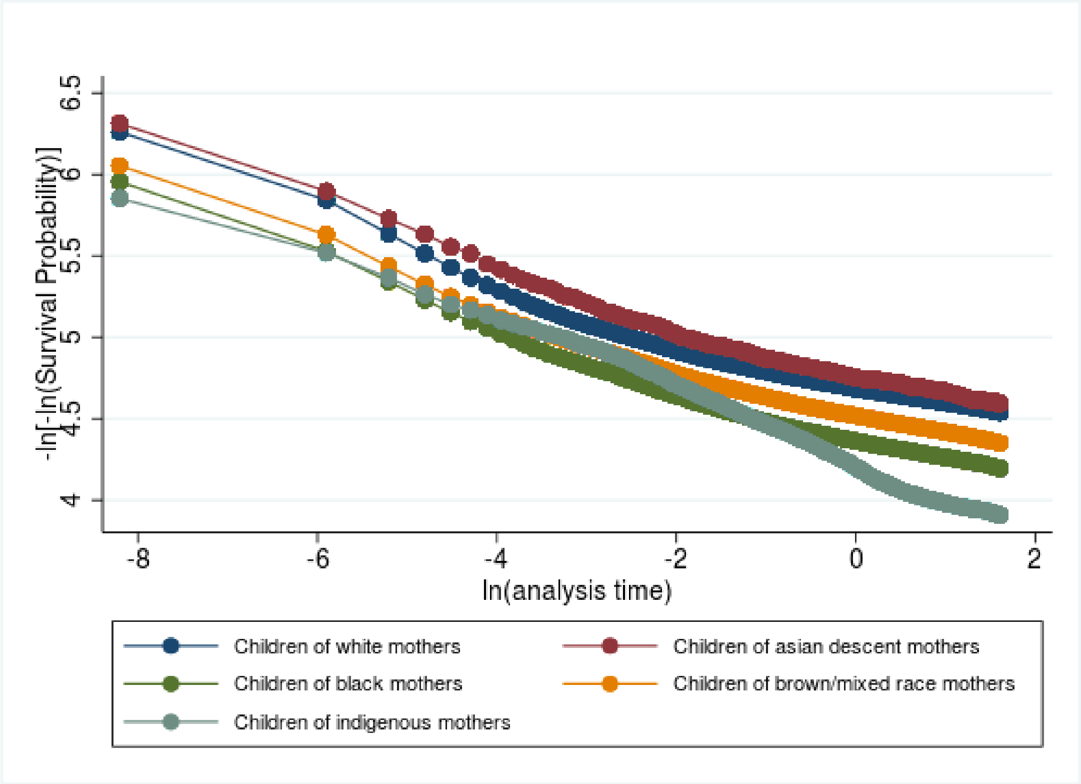

Table S3. Observational Routinely-Collected health Data (RECORD) guideline

The RECORD statement – checklist of items, extended from the STROBE statement, that should be reported in observational studies using routinely collected health data.

	**Item No.**	**STROBE items**	**Location in manuscript where items are reported**	**RECORD items**	**Location in manuscript where items are reported**
**Title and abstract**					
	1	(a) Indicate the study's design with a commonly used term in the title or the abstract (b) Provide in the abstract an informative and balanced summary of what was done and what was found	Abstract	RECORD 1.1: The type of data used should be specified in the title or abstract. When possible, the name of the databases used should be included.<br><br>RECORD 1.2: If applicable, the geographic region and timeframe within which the study took place should be reported in the title or abstract.<br><br>RECORD 1.3: If linkage between databases was conducted for the study, this should be clearly stated in the title or abstract.	Abstract
**Introduction**					
Background rationale	2	Explain the scientific background and rationale for the investigation being reported	Introduction: 1st to 4th paragraphs		Introduction: 1st to 4th paragraphs
Objectives	3	State specific objectives, including any prespecified hypotheses	Introduction: last paragraph		Introduction: last paragraph
**Methods**					
Study Design	4	Present key elements of study design early in the paper	Methods section: 1st paragraph - Study design and population (page 5)		Methods section: 1st paragraph - Study design and population (page 5)
Setting	5	Describe the setting, locations, and relevant dates, including periods of recruitment, exposure, follow-up, and data collection	Methods section: Study design and population (page 5) and Main expousure variable (pages 5 and 6)		Methods section: Study design and population (page 5) and Main expousure variable (pages 5 and 6)
Participants	6	*(a) Cohort study* - Give the eligibility criteria, and the sources and methods of selection of participants. Describe methods of follow-up	Methods section: Study design and population (methods of selection of participants) (page 5), Data linkage	RECORD 6.1: The methods of study population selection (such as codes or algorithms used to identify subjects) should be listed in detail. If this is not possible, an explanation should be provided.	6.1: Methods section: Study design and population (page 6), Data linkage (page 7)<br><br>6.2: Methods section: Data linkage (CIDACS-RL tool) (page 7)

		Case-control study - Give the eligibility criteria, and the sources and methods of case ascertainment and control selection. Give the rationale for the choice of cases and controls<br>*Cross-sectional study* - Give the eligibility criteria, and the sources and methods of selection of participants<br><br>*(b) Cohort study* - For matched studies, give matching criteria and number of exposed and unexposed<br>*Case-control study* - For matched studies, give matching criteria and the number of controls per case	(methods of selection of participants) (page 7)	RECORD 6.2: Any validation studies of the codes or algorithms used to select the population should be referenced. If validation was conducted for this study and not published elsewhere, detailed methods and results should be provided.<br><br>RECORD 6.3: If the study involved linkage of databases, consider use of a flow diagram or other graphical display to demonstrate the data linkage process, including the number of individuals with linked data at each stage.	6.3: Methods section: Study design and population; Figure 1 (Results section)
Variables	7	Clearly define all outcomes, exposures, predictors, potential confounders, and effect modifiers. Give diagnostic criteria, if applicable.	Methods section: pages 5-7	RECORD 7.1: A complete list of codes and algorithms used to classify exposures, outcomes, confounders, and effect modifiers should be provided. If these cannot be reported, an explanation should be provided.	All definitions can be found in the Methods section: pages 5-7
Data sources/ measurement	8	For each variable of interest, give sources of data and details of methods of assessment (measurement).<br>Describe comparability of assessment methods if there is more than one group	Methods section: Study design and population (pages 5), Main exposure variable (page 5 and 6), Covariates (page 6), Outcomes (page 6), Statistical analysis (page 7 and 8)		Methods section: Study design and population (pages 5), Main exposure variable (page 5 and 6), Covariates (page 6), Outcomes (page 6), Statistical analysis (page 7 and 8)
Bias	9	Describe any efforts to address potential sources of bias	Covariates (page 6), Analysis (pages 7-8)		Covariates (page 6), Analysis (pages 7-8)
Study size	10	Explain how the study size was arrived at	Figure 1 (Results section)		Figure 1 (Results section)
Quantitative variables	11	Explain how quantitative variables were handled in the analyses. If applicable, describe which groupings were chosen, and why	Table 1 (Results section)<br>Information regarding these variables can be found throughout the methods: Covariates (page 6)		Table 1 (Results section)<br>Information regarding these variables can be found throughout the methods: Covariates (page 6)
Statistical methods	12	(a) Describe all statistical methods, including those used to control for confounding<br>(b) Describe any methods used to examine subgroups and interactions	Methods section: Statistical Analysis (pages 7-8)		Methods section: Statistical Analysis (pages 7-8)

		(c) Explain how missing data were addressed<br>(d) *Cohort study* - If applicable, explain how loss to follow-up was addressed<br>*Case-control study* - If applicable, explain how matching of cases and controls was addressed<br>*Cross-sectional study* - If applicable, describe analytical methods taking account of sampling strategy<br>(e) Describe any sensitivity analyses			
Data access and cleaning methods		..	Methods section: Study design and population (pages 5), Main exposure variable (page 5 and 6), Covariates (page 6), Outcomes (pages 6 and 7), Data linkage (page 6), Statistical Analysis (pages 7 and 8).<br>Results section: Figure 1	RECORD 12.1: Authors should describe the extent to which the investigators had access to the database population used to create the study population.<br><br>RECORD 12.2: Authors should provide information on the data cleaning methods used in the study.	RECORD 12.1: Methods section: Study design and population (pages 5), Main exposure variable (page 5 and 6), Covariates (page 6), Outcomes (pages 6 and 7), Data linkage (page 6), Statistical Analysis (pages 7 and 8).<br><br>RECORD 12.2:<br>Methods section: Study design and population (page 5)<br>Results section: Figure 1
Linkage		..		RECORD 12.3: State whether the study included person-level, institutional-level, or other data linkage across two or more databases. The methods of linkage and methods of linkage quality evaluation should be provided.	Methods section: Study design and population (page 5), Data linkage (page 6), Statistical Analysis (pages 7-8).
**Results**					
Participants	13	(a) Report the numbers of individuals at each stage of the study (*e.g.*, numbers potentially eligible, examined for eligibility, confirmed eligible, included in the study, completing follow-up, and analysed)<br>(b) Give reasons for non-participation at each stage.<br>(c) Consider use of a flow diagram	Method section: Study design and population 1st paragraph;<br>Figure 1	RECORD 13.1: Describe in detail the selection of the persons included in the study (*i.e.,* study population selection) including filtering based on data quality, data availability and linkage. The selection of included persons can be described in the text and/or by means of the study flow diagram.	Method section: Study design and population 1st paragraph;<br>Figure 1
Descriptive data	14	(a) Give characteristics of study participants (*e.g.*, demographic, clinical, social) and information on exposures and potential confounders	Table 1		Table 1

		(b) Indicate the number of participants with missing data for each variable of interest<br>(c) *Cohort study* - summarise follow-up time (*e.g.*, average and total amount)			
Outcome data	15	*Cohort study* - Report numbers of outcome events or summary measures over time<br>*Case-control study* - Report numbers in each exposure category, or summary measures of exposure<br>*Cross-sectional study* - Report numbers of outcome events or summary measures	Results section: 5th paragraph;<br>Table 2		Results section: 5th paragraph;<br>Table 2
Main results	16	(a) Give unadjusted estimates and, if applicable, confounder-adjusted estimates and their precision (e.g., 95% confidence interval). Make clear which confounders were adjusted for and why they were included<br>(b) Report category boundaries when continuous variables were categorized<br>(c) If relevant, consider translating estimates of relative risk into absolute risk for a meaningful time period	Results section (6th to 9th paragraph)<br>Tables 2 and 3		Results section (6th to 9th paragraph)<br>Tables 2 and 3
Other analyses	17	Report other analyses done—e.g., analyses of subgroups and interactions, and sensitivity analyses	Results section (last paragraph)<br>Supplementary Material: Tables S2-S3		Results section (last paragraph)<br>Supplementary Material: Tables S2-S3
**Discussion**					
Key results	18	Summarise key results with reference to study objectives	Discussion section (1st paragraph)		Discussion section (1st paragraph)
Limitations	19	Discuss limitations of the study, taking into account sources of potential bias or imprecision. Discuss both direction and magnitude of any potential bias	Discussion section (9th paragraph)	RECORD 19.1: Discuss the implications of using data that were not created or collected to answer the specific research question(s). Include discussion of misclassification bias, unmeasured confounding, missing data, and changing eligibility over time, as they pertain to the study being reported.	Discussion section<br>(9th paragraph)
Interpretation	20	Give a cautious overall interpretation of results considering objectives, limitations, multiplicity of analyses, results from similar studies, and other relevant evidence	Discussion section: page 12 to 14		Discussion section: page 12 to 14

Generalisability	21	Discuss the generalisability (external validity) of the study results	Discussion section: page 12 (2nd, 4th paragraphs)		Discussion section: page 12 (2nd, 4th paragraphs)
**Other Information**					
Funding	22	Give the source of funding and the role of the funders for the present study and, if applicable, for the original study on which the present article is based	Funding (page 8)		Funding (page 8)
Accessibility of protocol, raw data, and programming code		..	Methods section: Data linkage	RECORD 22.1: Authors should provide information on how to access any supplemental information such as the study protocol, raw data, or programming code.	Methods section: Data linkage

*Reference: Benchimol EI, Smeeth L, Guttmann A, Harron K, Moher D, Petersen I, Sørensen HT, von Elm E, Langan SM, the RECORD Working Committee. The REporting of studies Conducted using Observational Routinely-collected health Data (RECORD) Statement. *PLoS Medicine* 2015; in press.

*Checklist is protected under Creative Commons Attribution (CC BY) license.

Table S4. Crude and adjusted Hazard Ratio for the association between maternal race/skin colour and neonatal mortality, according to the main causes of death.

Main causes of death		Children of white mothers	Children of indigenous mothers	Children of black mothers	Children of brown/mixed mothers	Children of asian descent mothers
Diarrhoea	**N=145**	**N=29**	**N=7**	**N=10**	**N=99**	**N=0**
	**Death/100,000 PYR (95% CI)**	96.23 (88.37-104.80)	2976 (285.09-3300)	208.06 (179.10-241.71)	229.63 (219.55-240.17)	82.52 (34.35-198.26)
	**HR (95% CI)**	1.00	30.98 (27.10 - 35.42)	2.16 (1.82 - 2.57)	2.39 (2.17 - 2.63)	0.86 (0.35 - 2.07)
	**HR[1] (95% CI)**	1.00	14.16 (12.15 - 16.51)	1.71 (1.43 - 2.03)	1.78 (1.61 - 1.98)	0.80 (0.33 - 1.93)
Flu and pneumonia	**N=415**	**N=102**	**N=24**	**N=26**	**N=262**	**N=1**
	**Death/100,000 PYR (95% CI)**	325.70 (310.95-341.16)	3710 (3383-4069)	677.73 (623.72-736.41)	638.16 (621.21-655.58)	445.61 (305.59-649.78)
	**HR (95% CI)**	1.00	11.41 (10.29 – 12.65)	2.08 (1.89 - 2.29)	1.96 (1.86 - 2.07)	1.37 (0.94 - 2.00)
	**HR[1] (95% CI)**	1.00	6.43 (5.73 - 7.21)	1.77 (1.61 - 1.95)	1.60 (1.51 - 1.69)	1.32 (0.90 - 1.93)
Malnutrition	**N=45**	**N=5**	**N=4**	**N=1**	**N=35**	**N=0**
	**Death/100,000 PYR (95% CI)**	29.89 (25.65-34.83)	1542 (1336-1779)	102.21 (82.53-126.58)	97.95 (91.44-104.92)	33.01 (8.26-131.98)
	**HR (95% CI)**	1.00	51.68 (41.90 - 63.73)	3.42 (2.63 - 4.45)	3.28 (2.77 - 3.88)	1.11 (0.27 - 4.46)
	**HR[1] (95% CI)**	1.00	16.24 (12.77 - 20.67)	2.33 (1.77 - 3.05)	2.04 (1.71 - 2.45)	1.11 (0.27 - 4.46)
Selected accidental causes[2]	**N=24**	**N=0**	**N=4**	**N=0**	**N=15**	**N=5**
	**Death/100,000 PYR (95% CI)**	96.78 (88.89-105.37)	420.55 (319.61-553.36)	150.87-126.52-1709.91)	153.48 (145.28-162.15)	82.52 (34.34-198.25)
	**HR (95% CI)**	1.00	4.35 (3.26-5.80)	1.56 (1.28-1.89)	1.58 (1.43-1.75)	0.85 (0.35-2.05)
	**HR[1] (95% CI)**	1.00	1.71 (1.25-2.34)	1;36 (1.11-1;67)	1.17 (1.04-1.32)	0.82 (0.34-1.99)
Ill-defined causes	**N=894**	**N=202**	**N=48**	**N=48**	**N=595**	**N=1**
	**Death/100,000 PYR (95% CI)**	251.89 (238.95-265.52)	1995 (1759-2263)	681.38 (627.22-740.22)	482.75 (468.03-497.93)	330.08 (212.95-511.63)3,3
	**HR (95% CI)**	1.00	7.93 (6.92 - 9.09)	2.71 (2.45 - 2.99)	1.92 (1.80 - 2.04)	1.31 (0.84 - 2.04)
	**HR[1] (95% CI)**	1.00	4.22 (3.64 - 4.90)	2.28 (2.06 - 2.52)	1.57 (1.48 - 1.68)	1.28 (0.82 - 1.99)

[1] Model adjusted for the variables: region, mother's education, year of birth.
[2]The selected accidental causes refer to deaths from drowning and falls.
PYR: person-year at risk

Table S5. Crude and adjusted Hazard Ratio for the association between maternal race/skin colour and 1 to 4 years old mortality, according to the main causes of death.

Main causes of death		Children of white mothers	Children of indigenous mothers	Children of black mothers	Children of brown/mixed mothers	Children of asian descent mothers
Diarrhoea	**N=616**	**N=99**	**N=144**	**N=31**	**N=342**	**N=0**
	**Death/100,000 PYR (95% CI)**	0.63 (0.51-0.76)	42.13 (35.80-49.58)	1.28 (0.90-1.83)	1.42 (1.27-1.57)	0
	**HR (95% CI)**	1.00	66.51 (51.71 - 85.56)	2.05 (1.37 - 3.05)	2.25 (1.81 - 2.80)	0
	**HR[1] (95% CI)**	1.00	34.12 (25.53 - 45.59)	1.63 (1.09 - 2.46)	1.80 (1.43 - 2.27)	0
Flu and pneumonia	**N=2336**	**N=596**	**N=100**	**N=142**	**N=1489**	**N=9**
	**Death/100,000 PYR (95% CI)**	3.70 (3.42-4.01)	29.06 (23.88-35.35)	5.96 (5.06-7.02)	6.23 (5.92-6.54)	5.63 (3.03-10.46)
	**HR (95% CI)**	1.00	7.77 (6.29 - 9.60)	1.61 (1.34 - 1.93)	1.67 (1.52 - 1.84)	1.52 (0.81 - 2.84)
	**HR[1] (95% CI)**	1.00	5.29 (4.20 - 6.66)	1.46 (1.22 - 1.75)	1.47 (1.32 - 1.62)	1.49 (0.80 - 2.79)
Malnutrition	**N=295**	**N=40**	**N=74**	**N=17**	**N=163**	**N=1**
	**Death/100,000 PYR (95% CI)**	0.24 (0.17-0.33)	21.50 (17.12 - 27.00)	0.75 (0.46-1.18)	0.71 (0.61-0.81)	0.56 (0.07-3.99)
	**HR (95% CI)**	1.00	88.19 (60.03 - 129.56)	3.08 (1.77 - 5.38)	2.90 (2.06 - 4.10)	2.33 (0.32 - 16.98)
	**HR[1] (95% CI)**	1.00	36.08 (23.24 - 56.02)	2.42 (1.38 - 4.25)	2.23 (1.55 - 3.20)	2.26 (0.31 - 16.45)
Selected accidental causes[2]	**N=1547**	**N=432**	**N=35**	**N=87**	N=988	**N=5**
	**Death/100,000 PYR (95% CI)**	2.72 (2.48-2.99)	10.46 (7.54-14.50)	3.76 (3.06-4.62)	4.32 (4.07-4.59)	2.81 (1.17-6.76)
	**HR (95% CI)**	1.00	3.82 (2.72-5.36)	1.37 (1.10-1.72)	1.58 (1.41-1.76)	1.03 (0.42-2.49)
	**HR[1] (95% CI)**	1.00	1.41 (0.98-2.02)	1.16 (0.92-1.46)	1.11 (0.97-1.27)	0.96 (0.40-2.33)
Ill-defined causes	**N=1184**	**N=244**	**N=66**	**N=112**	**N=755**	**N=7**
	**Death/100,000 PYR (95% CI)**	1.53 (1.35-1.73_	19.18 (15.06-24.41)	4.80 (4.00-5.76)	3.28 (3.05-3.51)	3.93 (1.87-8.26)
	**HR (95% CI)**	1.00	12.45 (9.50 - 16.32)	3.13 (2.52 - 3.90)	2.13 (1.85 - 2.46)	2.57 (1.21 - 5.45)
	**HR[1] (95% CI)**	1.00	6.29 (4.66 - 8.48)	2.60 (2.08 - 3.24)	1.63 (1.40 - 1.90)	2.43 (1.15 - 5.16)

[1]Model adjusted for the variables: region, mother's education, year of birth.
[2]The selected accidental causes refer to deaths from drowning and falls.
PYR: person-year at risk

Figure S3 - Cumulative under-five mortality per 100 000 person-years at risk by maternal race/skin colour obtained by the Nelson-Aalen method

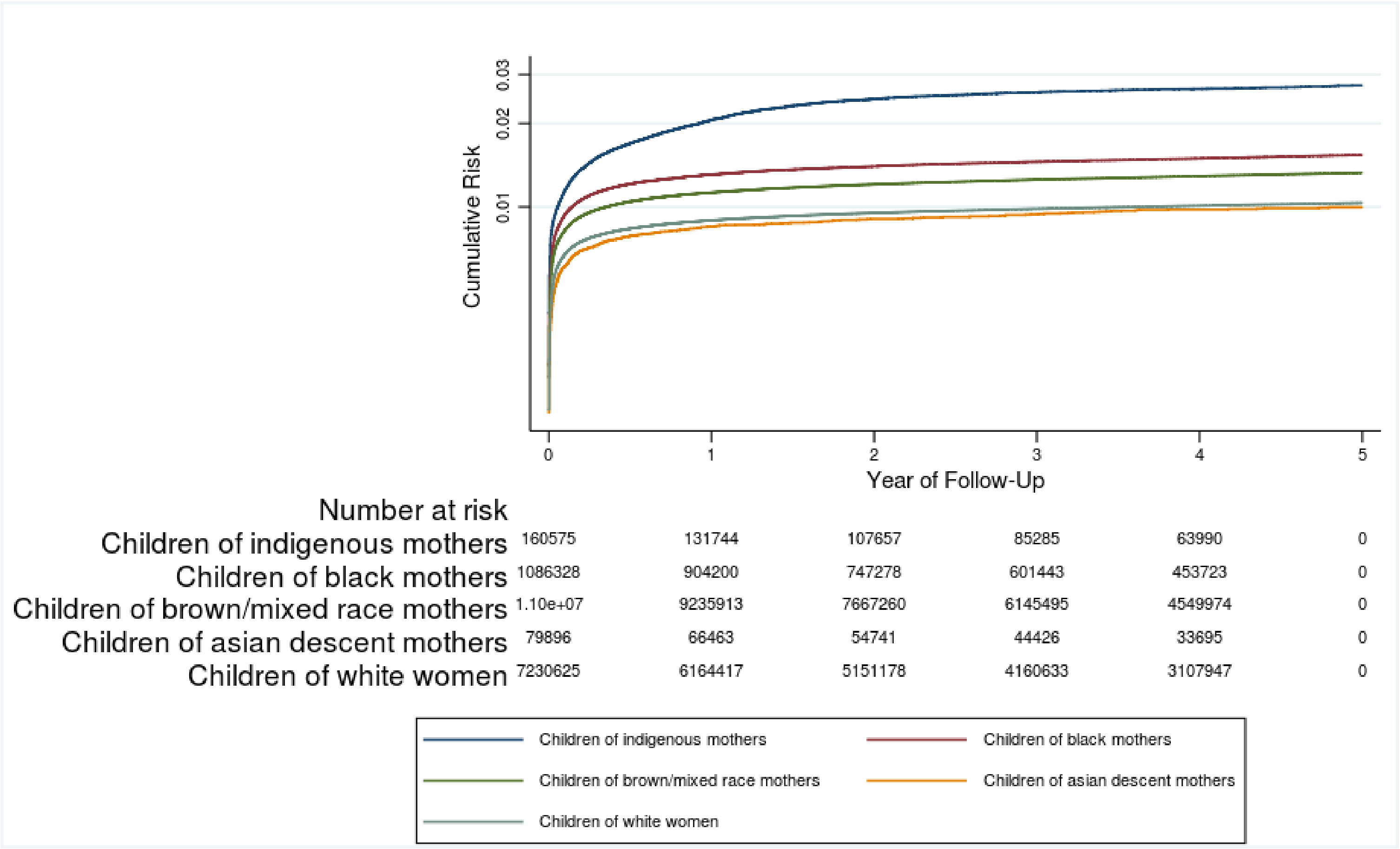

Table S6. Crude and adjusted Hazard Ratio for the association between maternal race/skin colour and mortality by age group, with a finite population correction.

Age groups		Children of white mothers	Children of indigenous mothers	Children of black mothers	Children of brown/mixed mothers	Children of asian descent mothers
Under-five years	**N=224,213**	**N=69,880 (31%)**	**N=3874 (2%)**	**N=15,393 (7%)**	**N=134,331 (60%)**	**N=735 (0.3%)**
	**Death/100,000 PYR**	300.40 (298.18-302.64)	792.51 (767.94-817.86)	452.32 (445.23-459.52)	388.63 (386.56-390.72)	293.39 (272.92-315.38)
	**HR (95% CI)**	1.00	2.53 (2.45 - 2.60)	1.48 (1.45 - 1.50)	1.27 (1.26 - 1.28)	0.96 (0.90 - 1.02)
	**HR[1] (95% IC)**	1.00	1.98 (1.92 - 2.04)	1.39 (1.36 - 1.41)	1.19 (1.18 - 1.20)	0.94 (0.88 - 1.00)
1 to 4 years	**N=22,196**	**N=6345 (29%)**	**N=715 (3%)**	**N=1528 (7%)**	**N=13,539 (61%)**	**N=71 (0.3%)**
	**Death/100,000 PYR**	38.24 (37.31-39.19)	207.77 (193.08-223.57)	63.28 (60.19-66.54)	55.19 (54.27-56.13)	39.96 (31.67-50.43)
	**HR (95% IC)**	1.00	5.40 (5.05 - 5.78)	1.65 (1.57 - 1.73)	1.44 (1.40 - 1.48)	1.05 (0.82 - 1.29)
	**HR[1] (95% IC)**	1.00	3.83 (3.55 - 4.11)	1.50 (1.43 - 1.58)	1.30 (1.26 - 1.34)	1.00 (0.81 - 1.23)
Under 1 year	**N=202,012**	**N=63,535 (32%)**	**N=3159 (2%)**	**N=13,864 (7%)**	**N=120,790 (60%)**	**N=664 (0.3%)**
	**Death/100,000 PYR**	950.98 (943.61-958.40)	2179 (2104-2256)	1399 (1376-1423)	1201 (1194-1208)	909.69 (843.07-981.58)
	**HR (95% CI)**	1.00	2.25 (2.18 - 2.33)	1.46 (1.43 - 1.48)	1.26 (1.25 - 1.27)	0.94 (0.87 - 1.02)
	**HR[1] (95% CI)**	1.00	1.80 (1.74 - 1.86)	1.37 (1.35 - 1.40)	1.18 (1.17 - 1.19)	0.85 (0.78 - 0.92)
Postneonatal (28 to 364 days)	**N=58,344**	**N=17,165 (29%)**	**N=1455 (3%)**	**N=4280 (7%)**	**N=35247 (60%)**	**N=197 (0.3%)**
	**Death/100,000 PYR**	279.02 (274.88-283.23)	1091 (1037-1149)	469.69 (455.82-483.97)	380.85 (376.89-384.85)	293.38 (255.15-337.35)
	**HR (95% CI)**	1.00	3.88 (3.71 - 4.07)	1.68 (1.63 - 1.73)	1.36 (1.34 - 1.38)	1.05 (0.92 - 1.18)
	**HR[1] (95% CI)**	1.00	2.79 (2.65 - 2.93)	1.54 (1.23 - 1.27)	1.25 (1.23 - 1.27)	1.03 (0.91 - 1.17)
Neonatal (27 days)	**N=143,668**	**N=46,370 (32%)**	**N=1704 (1%)**	**N=9584 (7%)**	**N=85,543 (60%)**	**N=467 (0.3%)**
	**Death/100,000 PYR**	8451 (8374-8528)	14,051 (13,399-14,734)	11,661 (11,430-11,897)	10,305 (10,237-10,375)	7707 (7039-8439)
	**HR (95% CI)**	1.00	1.66 (1.59 - 1.73)	1.38 (1.35 - 1.40)	1.22 (1.21 - 1.23)	0.91 (0.84 - 0.99)
	**HR[1] (95% CI)**	1.00	1.38 (1.31 - 1.44)	1.31 (1.29 - 1.34)	1.16 (1.14 - 1.17)	0.89 (0.82 - 0.97)

[1]Model adjusted for the variables: region, mother's education, year of birth.
PYR: person-year at risk
